# Steroid Biosynthesis Pathway Counteracts Iron Overload-Induced Ferroptosis in Mouse Granulosa Cells

**DOI:** 10.3390/biology15141182

**Published:** 2026-07-17

**Authors:** Feiyan Gao, Weiran Mao, Xiaoying He, Ying Liu, Yang Liu, Shujun Liu, Jiwei Liu, Libing Ma

**Affiliations:** 1 School of Life Science and Technology, Inner Mongolia University of Science and Technology, Baotou 014010, China; gfy2868@163.com (F.G.); 19156186806@163.com (W.M.); hxy1124@163.com (X.H.); liuying1529@163.com (Y.L.); yangl1991@163.com (Y.L.); liushujun92@163.com (S.L.); 2 Inner Mongolia Key Laboratory of Life Health and Bioinformatics, Inner Mongolia University of Science and Technology, Baotou 014010, China

**Keywords:** iron overload, Ferroptosis, steroid biosynthesis, granulosa cells, SREBF2, female infertility

## Abstract

Iron overload is a recognized risk factor for female reproductive dysfunction, yet the underlying cellular and molecular mechanisms remain incompletely understood. In this study, the effects of iron overload on ovarian granulosa cells were investigated, and a protective role of the steroid biosynthesis pathway against ferroptosis was identified. A mouse model of ovarian iron overload was established by daily gavage of ferric citrate (FC, 120 mg/kg for 40 days). Iron-overloaded female mice exhibited disrupted estrous cycles, reduced serum estradiol levels, impaired antral follicle development, and decreased pregnancy rates and litter sizes. Metabolomic analysis of freshly isolated granulosa cells revealed significant depletion of unsaturated glycerophospholipids and fatty acids, along with reduced antioxidants such as glutathione, vitamin E, and coenzyme Q6, and enrichment of the ferroptosis pathway. Transcriptomic analysis showed marked upregulation of genes involved in steroid biosynthesis, including *Hmgcr* and *Fdft1*, and their master transcription factor *Srebf2*. In cultured KK1 granulosa cells, FC treatment increased intracellular Fe^2+^ and reactive oxygen species, decreased glutathione content and NADPH/NADP^+^ ratio, elevated malondialdehyde levels, and induced lipid peroxidation and plasma membrane rupture, all of which were attenuated by the iron chelator deferoxamine. Knockdown of *Srebf2* suppressed *Hmgcr* and *Fdft1* expression, exacerbated lipid peroxidation, and increased membrane damage in iron-overloaded cells, confirming that SREBF2-driven steroid biosynthesis acts as an endogenous anti-ferroptotic mechanism. Collectively, these findings demonstrate that iron overload triggers ferroptosis in granulosa cells, leading to follicular arrest and reduced fertility, and that activation of the steroid biosynthesis pathway counteracts ferroptosis, likely through the production of protective intermediates. This study provides a mechanistic basis for iron overload-induced female infertility and identifies the steroid biosynthesis pathway as a potential therapeutic target.

## Simple Summary

Iron is essential for health, but too much iron in the body can damage the ovaries and cause infertility. In this study, how excess iron harms ovarian cells in mice was investigated. It was discovered that iron overload causes a specific type of cell death called ferroptosis in granulosa cells—the cells that support egg development and produce the hormone estrogen. This cell death reduces estrogen levels, disrupts the normal reproductive cycle, and lowers fertility. Interestingly, it was discovered that these damaged cells try to protect themselves by activating a cholesterol-producing pathway. However, this self-defence mechanism is not strong enough to fully prevent cell death. These findings explain, at the molecular level, why iron overload leads to infertility, and they suggest that strengthening this protective pathway could become a new strategy to preserve fertility in women with iron overload conditions—such as those with thalassaemia or those exposed to high levels of iron through their occupation. This research opens new possibilities for developing treatments to protect ovarian function in at-risk women.

## 1. Introduction

Female infertility has evolved into a significant global public health concern [1]. Emerging evidence has identified iron overload as a clinically relevant reproductive toxicant that compromises ovarian function. Occupationally, workers in mining, milling, and welding industries are at risk of systemic iron accumulation through inhalation, skin contact, and consumption of contaminated water and food [2,3,4]. Clinically, patients with transfusion-dependent β-thalassaemia major represent a well-characterized population in which ovarian iron deposition directly contributes to gonadotoxicity and subfertility [5,6]. Although iron is essential for normal cellular metabolism, its deficiency has long been recognized for its adverse effects on the organism and the various diseases it triggers, such as anemia and growth arrest [7,8]. Conversely, its excess drives the Fenton reaction and disrupts redox homeostasis, causing oxidative damage to multiple tissues [9,10,11,12,13]. Within the ovary, iron overload has been shown to impair folliculogenesis, oocyte quality, and steroidogenesis [5,6,14,15,16,17,18,19]. However, the specific cellular and molecular mechanisms through which ovarian iron overload undermines female fertility remain incompletely understood, necessitating further mechanistic investigation.

Iron overload has been consistently shown to impair oocyte development and reproductive capacity in female mice [14,15]. Among women seeking fertility treatment, daily supplemental iron intake exceeding 45 mg is significantly associated with a lower antral follicle count [16]. In patients with transfusion-dependent β-thalassaemia major, the clinical severity of iron overload is paralleled by reduced antral follicle counts, diminished ovarian reserve, and impaired fertility outcomes [5,6,17,18,19]. However, most studies in this area have focused on documenting these phenotypic changes—such as reduced estrogen levels and decreased follicle numbers—without investigating the underlying molecular mechanisms [5,16,17,18].

Granulosa cells, the principal site of oestrogen biosynthesis, require a steady supply of cholesterol and the coordinated activity of enzymes such as steroidogenic acute regulatory protein (StAR) and aromatase (CYP19A1) to sustain the steroidogenic cascade [20]. Despite the established link between iron overload and reduced oestrogen synthesis [5,14,17,18], the specific molecular events through which excess iron perturbs this steroidogenic machinery in granulosa cells remain largely unexplored. To address this mechanistic gap, we established a mouse model of ovarian iron overload and performed systematic, unbiased metabolomic and transcriptomic profiling on freshly isolated granulosa cells—with the explicit aim of comprehensively surveying, from both the metabolic and gene-expression levels, the molecular alterations responsible for iron-impaired oestrogen production. These multi-omics analyses yielded two main findings. First, iron overload triggered ferroptosis in granulosa cells, as evidenced by depletion of unsaturated glycerophospholipids (GPs), glutathione (GSH), vitamin E (VE), and coenzyme Q6 (CoQ6), suggesting that ferroptosis may represent a key mechanism underlying iron-induced impairment of oestrogen synthesis and fertility. Second, iron overload markedly upregulated the genes in the cholesterol biosynthetic pathway, including *Hmgcr* and *Fdft1*, under the control of their master transcription factor *Srebf2*, raising the possibility that activation of this pathway serves to generate protective intermediates that counteract ferroptosis. To validate these findings and test this functional hypothesis, we established an in vitro iron-overloaded granulosa cell model using the mouse KK1 cell line and performed targeted gene knockdown experiments. Collectively, this study provides a mechanistic framework linking iron overload to granulosa cell ferroptosis and female infertility, and identifies the steroid biosynthesis pathway as a potential therapeutic target for fertility preservation in iron overload-related conditions.

## 2. Materials and Methods

### 2.1. Ferric Citrate Gavage in Mice and Detection of Ovarian Iron Content

C57BL/6J female mice were purchased from Beijing HFK Bioscience Co., Ltd. (Beijing, China). At 6 weeks of age, the mice were randomly divided into four groups and administered daily either saline or ferric citrate (FC, Sigma-Aldrich, Merck, Shanghai, China) at doses of 60, 120, or 180 mg/kg by gavage for up to 50 consecutive days.

During the gavage period, 3 mice from each group were euthanized by cervical dislocation every 10 days, and ovarian tissues were collected. Ovarian iron content was measured using a tissue iron assay kit (Solarbio, Beijing, China) according to the manufacturer’s instructions. Ovarian iron overload model mice were defined as those in which ovarian Fe^2+^ content in the FC-treated groups was significantly higher (*p* < 0.05) than that in the control group; these mice were used for subsequent experiments.

The overall experimental design, mouse number, and allocation are summarized in Supplementary Figure S1. Briefly, a total of 380 female C57BL/6J mice (6 weeks old) were purchased in three separate batches for different experimental purposes. Batch 1 (*n* = 100) was used to monitor ovarian iron content over time and to establish the iron overload model. Batch 2 (*n* = 80) was used for assessments of serum estradiol (E2) levels, ovarian histology, estrous cycle, and fertility. Batch 3 (*n* = 200) was used for granulosa cell collection and subsequent metabolomic and transcriptomic analyses.

### 2.2. Detection of the Estrous Cycle in Mice

Vaginal secretions were collected daily from ten mice in each of the control and iron overload groups at a fixed time (9:00–10:00 a.m.) for 20 consecutive days. The secretions were gently smeared onto glass slides, air-dried, and examined directly under an optical microscope (E100, Nikon, Tokyo, Japan) without staining. The stage of the estrous cycle (diestrus, proestrus, estrus, or metestrus) was determined based on the predominant cell types (neutrophils, epithelial cells, or cornified epithelial cells) present in the smears, according to the criteria described by Maurya et al. [21]. Blinding procedures for subjective assessments were not performed in this study.

### 2.3. Detection of Pregnancy Rate and Litter Size in Mice

Nine mice from each of the control and the iron overload groups were co-housed with male mice at a female-to-male ratio of 3:1. After 7 days of co-housing, the male mice were removed, and the pregnancy status of the female mice was monitored and recorded. Approximately 21 days after co-housing, the number of pups per female was recorded at birth.

### 2.4. Detection of Serum Sex Hormone Levels in Mice

Ten mice in each of the control and the iron overload groups were euthanized by cervical dislocation. Eyeballs were enucleated using curved forceps, and blood was collected into 1.5 mL centrifuge tubes. The blood samples were allowed to stand at 4 °C for 24 h, followed by centrifugation at 4 °C and 3000 rpm for 10 min using a TGL-18M high-speed refrigerated centrifuge (Bioridge, Shanghai, China) with a No. 2 fixed-angle rotor. The upper serum layer was collected and stored at −20 °C for subsequent measurement of sex hormone levels.

Serum level of E2 was measured using a Mouse Estradiol (E2) ELISA Kit (Dogesce, Beijing, China), in conjunction with a microplate reader (Synergy HT, BioTek Instruments, Inc., Winooski, VT, USA). All procedures were performed according to the manufacturers’ instructions.

### 2.5. Ovarian Paraffin Section Preparation and HE Staining

Ovarian tissues from ten mice in each of the control and the iron overload groups were collected after cervical dislocation. The tissues were immediately fixed in 4% (*m*/*v*) paraformaldehyde for 24 h. After fixation, the tissues were dehydrated through a graded ethanol series: 70%, 80%, 90%, and 100% (*v*/*v*) ethanol, for 30 min each, to prevent brittleness.

Thereafter, the tissues were cleared in xylene for 15 min, infiltrated with two changes in molten paraffin for 1 h each, and embedded in paraffin blocks. Sections of 4–6 μm thickness were cut using a microtome (RM2235, Leica Biosystems, Nussloch, Germany), mounted on glass slides, and dried at 60 °C for 30 min.

For deparaffinization and rehydration, the sections were placed in xylene for 5 min twice, then rehydrated through a descending ethanol series (100%, 90%, 80%, and 70% (*v*/*v*)) for 2 min each, followed by a rinse in tap water for 5 min.

For hematoxylin and eosin (HE) staining, the sections were stained with hematoxylin (ready-to-use working solution, Solarbio) for 5 min, rinsed in tap water, differentiated in 1% hydrochloric acid in 70% ethanol for 3–5 s, and blued in running tap water. Thereafter, the sections were stained with eosin (ready-to-use working solution, Solarbio) for 5 min and then briefly dipped in tap water for 2–3 s.

After staining, the sections were dehydrated through an ascending ethanol series (70%, 80%, 90%, and 100% (*v*/*v*)) for 2 min each, cleared in xylene for 5 min twice, and mounted with neutral balsam. Finally, the sections were observed and photographed under an ultra-high-resolution microscope (DM3000 LED, Leica Microsystems CMS GmbH, Wetzlar, Germany).

### 2.6. Isolation of Granulosa Cells from the Ovary

Mice in the control group and the iron overload group were intraperitoneally injected with 10 IU per mouse of pregnant mare serum gonadotropin (PMSG, dissolved in sterile physiological saline) (Ningbo Second Hormone Factory, Ningbo, China). At 36 h after PMSG injection, the mice were euthanized by cervical dislocation, and the ovaries were collected. Ovarian granulosa cells were then isolated using the method reported by Tian et al. [22] for subsequent experiments.

### 2.7. Metabolomic Analysis of Granulosa Cells

Freshly isolated granulosa cells from the control and iron-overload groups were rapidly washed 2–3 times with pre-chilled PBS at 4 °C, then collected by centrifugation at 1000× *g* for 10 min using a TGL-18M high-speed refrigerated centrifuge with a No. 3 fixed-angle rotor. For each group, granulosa cells isolated from nine female mice were pooled as a single biological replicate, and a total of seven such biological replicates per group were submitted to Metware Biotechnology Inc. (Wuhan, China) for untargeted metabolomics analysis.

Cell samples were extracted with 80% methanol containing internal standards, subjected to three freeze–thaw cycles, and centrifuged. The supernatant was collected and analyzed using a Waters ACQUITY Premier HSS T3 column (1.8 µm, 2.1 mm × 100 mm, Waters Corporation, Milford, MA, USA) on a Thermo Scientific Q Exactive HF-X mass spectrometer (Thermo Fisher Scientific, Waltham, MA, USA) in both positive and negative ion modes.

Raw data were converted to mzML format using ProteoWizard and processed with eXtensible Computational Mass Spectrometry (XCMS, Scripps Research, San Diego, CA, USA) for peak detection, alignment, and retention time correction. Metabolites were identified against an in-house database combined with public libraries. Only compounds with identification score ≥0.5 and coefficient of variation (CV) < 0.3 in Quality Control (QC) samples were retained. QC samples were injected every 10 runs to monitor instrument stability.

Principal Component Analysis (PCA) and Orthogonal Partial Least Squares Discriminant Analysis (OPLS-DA) were performed using the MetaboAnalystR package (v1.0.1) (R software, www.r-project.org) on log2-transformed, mean-centered data. Differential metabolites were selected based on Variable Importance in Projection (VIP) > 1 and *p* < 0.05 (Student’s *t*-test). Kyoto Encyclopedia of Genes and Genomes (KEGG) pathway enrichment was conducted using hypergeometric testing (*p* < 0.05). Hierarchical clustering heatmaps were generated using the ComplexHeatmap package (v2.9.4) (R software) on unit variance scaling (UV-scaled) data.

### 2.8. RNA Sequencing

For the control and iron-overload groups, granulosa cells isolated from seven female mice per group were pooled, suspended in 1 mL of Trizol reagent (Takara, Beijing, China), and used as one biological replicate. Four such biological replicates were prepared for each group and submitted to Metware Biotechnology Inc. for transcriptomic analysis.

cDNA libraries were constructed and sequenced on the MGI platform (MGI Tech Co., Ltd., Shenzhen, China). Raw reads were filtered using fastp (v0.23.2) to remove adapter-containing reads, reads with >10% N bases, and reads with >50% low-quality bases (Q ≤ 20). Clean reads were aligned to the mouse reference genome GRCm39 (Ensembl v109) using HISAT2 (v2.2.1), with mapping rates > 95% for all samples. Gene counts were quantified using featureCounts (v2.0.3). Differential expression analysis was performed using DESeq2 (v1.38.3) with |log_2_(fold change)| ≥ 1 and adjusted *p* < 0.05 as the threshold. Gene Ontology (GO), KEGG and Reactome enrichment analyses were conducted using clusterProfiler (v4.6.0).

### 2.9. Cell Culture In Vitro and Treatment

The KK-1 mouse granulosa cell line was obtained from Wuhan Warner Bio Biotechnology Co., Ltd. (Wuhan, China), and has been cryopreserved in our laboratory upon receipt. The identity and functional characteristics of this cell line have been well documented in previous studies [23,24], and no contamination was observed during routine culture. After thawing, KK1 cells were cultured in high-glucose Dulbecco’s modified Eagle’s medium (DMEM) (Gibco, Thermo Fisher Scientific) supplemented with 10% (*v*/*v*) fetal bovine serum (Gibco), 100 IU/mL penicillin, and 0.1 mg/mL streptomycin at 37 °C in a humidified atmosphere containing 5% CO_2_. Cells were divided into three groups: (1) untreated control group (culture medium only), (2) FC treatment group (treated with 1 mM or 2 mM FC for 12 h or 24 h), and (3) FC combined with deferoxamine (DFO) treatment group (treated with 1 mM or 2 mM FC combined with 100 μM DFO (Macklin, Shanghai, China) for 12 h or 24 h). After processing the cells with a ferrous ion ELISA kit (Elabscience, Wuhan, China), the Fe^2+^ content in the cells was measured using a microplate reader (Synergy HT). The iron-overloaded cellular model was defined as KK1 cells in which intracellular Fe^2+^ content after FC treatment was significantly (*p* < 0.05) higher than that in the untreated control group.

### 2.10. Measurement of Reactive Oxygen Species (ROS) Levels

The three groups of cells (control, 2 mM FC, or 2 mM FC combined with 100 μM DFO treatment for 24 h) were stained with 10 μM of the ROS fluorescent probe 2′,7′-dichlorofluorescein diacetate (DCFH-DA, Beyotime, Shanghai, China) for 20 min in a humidified incubator at 37 °C in an atmosphere of 5% CO_2_. Subsequently, the fluorescence intensity of the cells was measured by flow cytometry (FACSCelesta, BD Biosciences, San Jose, CA, USA).

### 2.11. Measurement of GSH and Malondialdehyde (MDA) Levels and the NADPH/NADP^+^ Ratio

The three groups of cells were pretreated with ELISA kits for GSH (Perseebio, Beijing, China), MDA (Perseebio), and NADPH/NADP^+^ (Beyotime) following the manufacturers’ instructions. The absorbance values were then measured using a microplate reader (Synergy HT) to determine the content or ratio of the above substances in the three groups of cells.

### 2.12. Lipid Peroxide Detection

KK1 cells were cultured in 6-well culture plates. After treatment with 2 mM FC either alone or in combination with 100 μM DFO for 24 h, the cells were processed using a BDPY 581/591 C11 lipid peroxidation detection kit (Beyotime) according to the manufacturer’s instructions. The lipid peroxidation in the cells was examined using a Laser Scanning Confocal Microscope (A1R, Nikon).

### 2.13. Detection of Cell Membrane Integrity

The three groups of cells were stained with 0.4% (*m*/*v*) trypan blue (Beyotime) for 5 min at room temperature. Subsequently, the cells were observed and photographed under an inverted microscope (Ti-U, Nikon). The number of trypan blue-positive cells and the total number of cells were counted, and the proportion of trypan blue-positive cells was calculated.

### 2.14. Quantitative Real-Time Polymerase Chain Reaction (qRT-PCR)

The effect of iron overload on the expression levels of *Hmgcr*, *Fdft1*, and *Srebf2* in granulosa cells was examined using qRT-PCR. The primer sequences are listed in Table 1 and were synthesized by BiOligo (Shanghai, China). Cells were lysed with RNAiso Plus (Takara) for total RNA extraction. The concentration and purity of RNA were measured using a NanoDrop 2000 spectrophotometer (Thermo Fisher Scientific). The reverse transcription (RT) reactions were performed using PrimeScript RT reagent Kit with gDNA Eraser (Perfect Real Time) (Takara). For genomic DNA removal, the reaction mixture consisted of 2.5 µL 5× gDNA Eraser Buffer, 1.25 µL gDNA Eraser, X µL (X = 2000/RNA concentration (ng/μL)) total RNA, and (8.75-X) µL RNase-free water in a total volume of 12.5 µL. The reaction mixture was incubated at 42 °C for 2 min to eliminate genomic DNA. Subsequently, 1.25 µL PrimeScript RT Enzyme Mix I, 5 µL RT Primer Mix, 5 µL 5× PrimeScript Buffer 2, and 1.25 µL RNase-free water were added to the reaction mixture, resulting in a total volume of 25 µL. The RT mixture was incubated at 42 °C for 15 min, followed by 85 °C for 5 s to inactivate the reverse transcriptase. TransStart Tip Green qPCR SuperMix (TransGen Biotech, Beijing, China) was used for qPCR. qPCR reaction mixture consisted of 1 μL of the products of RT reaction, 0.6 μL forward primer (10 μM), 0.6 μL reverse primer (10 μM), 7.5 μL 2 × TransStart Tip Green qPCR SuperMix, 0.3 µL 50 × ROX Reference Dye, and 5 μL nuclease-free H_2_O in a total volume of 15 μL. qPCR reaction was performed by denaturation at 95 °C for 30 s, followed by 40 cycles of 95 °C for 5 s and 58 °C for 34 s using a real-time thermal cycler (7500 Real-Time PCR System; Applied Biosystems, Thermo Fisher Scientific). Melting curve analysis was performed at 95 °C for 15 s, 60 °C for 1 min, and 95 °C for 15 s to confirm the specificity of the PCR products. *Tubulin* was used as endogenous control gene; the level of *Hmgcr*, *Fdft1*, and *Srebf2* in the FC or FC combination with DFO treatment groups was normalized to that of the control group using the 2^−ΔΔCT^ method.

### 2.15. RNA Interference (RNAi)

Small-interfering RNA (siRNA) targeting *Srebf2* and negative control (NC) siRNA were synthesized by Genecarer (Xi’an, China) and dissolved in RNase-free H_2_O to a final concentration of 20 μM. The siRNA sequences used are listed in Table 2.

KK1 cells were seeded in 6-well culture plates and cultured in high-glucose DMEM. When the cells reached 70–80% confluence, the culture medium was discarded, and 125 μL of Opti-DMEM (Gibco) supplemented with 5 μL of double-stranded siRNA (20 μM) and 4 μL of Lipo8000 transfection reagent (Beyotime) was added. At 36 h post-transfection, *Srebf2* levels were analyzed by qRT-PCR to confirm siRNA specificity. qRT-PCR was also used to detect the levels of *Hmgcr* and *Fdft1* following *Srebf2* knockdown. At 12 h post-transfection, the siRNA-transfected cells were treated with 2 mM FC for 24 h, and the BDPY 581/591 C11 lipid peroxidation detection kit (Beyotime) was used to analyze the effect of *Srebf2* knockdown on the lipid peroxidation in the cells.

### 2.16. Statistical Analysis

All experiments were repeated at least three times. Data are presented as mean ± standard deviation (SD). All statistical comparisons were performed using the “One-Way Analysis of variance (ANOVA)” procedure in IBM SPSS Statistics 26, with only two groups selected for each comparison. Statistical significance was set at * *p* < 0.05. Significance levels are indicated as * *p* < 0.05, ** *p* < 0.01, and *** *p* < 0.001.

## 3. Results

### 3.1. FC Infusion Induces Iron Overload in Mouse Ovaries

To investigate whether excessive iron intake leads to ovarian iron overload in female mice, the mice were administered with normal saline, 60, 120, or 180 mg/kg FC via gavage. During the gavage period, 3 mice from each group were euthanized by cervical dislocation every 10 days, and iron content in the ovaries was measured using an iron assay kit.

**Figure 1:**
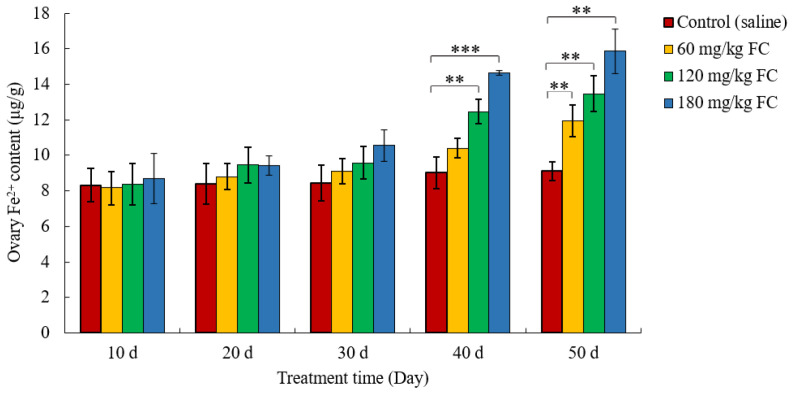
Effect of FC perfusion on Fe^2+^ content in mouse ovaries. (Female mice were perfused with saline or different concentrations of FC (60, 120, 180 mg/kg body weight). Ovarian Fe^2+^ content was measured every 10 days. The experiment was repeated three times, and data are presented as mean ± standard deviation. One-Way ANOVA was used to compare differences among groups. Significance is indicated as ** *p* < 0.01 and *** *p* < 0.001. A difference with *p* < 0.05 was considered statistically significant.).

As shown in Figure 1, continuous gavage with ≥120 mg/kg FC for 40 d resulted in significantly higher ovarian Fe^2+^ content compared with the control group. When the gavage period was extended to 50 d, even 60 mg/kg FC led to a significantly (*p* < 0.01) higher ovarian iron accumulation level than that in the control group. These results indicate that iron accumulation in the ovaries is both concentration- and time-dependent. Continuous gavage with 120 mg/kg FC for 40 d successfully established an ovarian iron overload mouse model for subsequent experiments.

### 3.2. Ovarian Iron Overload Disrupts the Estrous Cycle and Reduces Reproductive Capacity in Mice

To investigate the effect of iron overload on the estrous cycle in female mice, vaginal secretions were collected daily from control and iron-overloaded mice at fixed time points, smeared onto glass slides, and examined under a microscope. The estrous cycle stage of each mouse was determined according to the method described by Maurya et al. [21] and monitoring was continued for 20 consecutive days.

**Figure 2:**
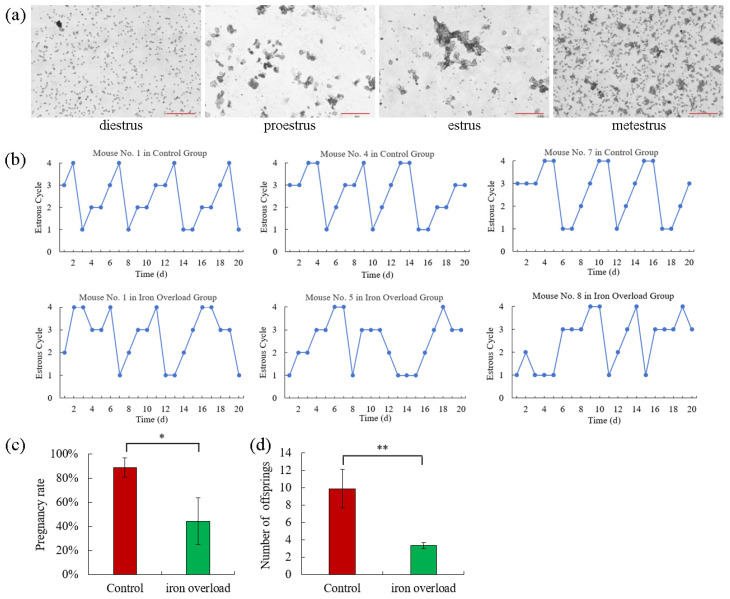
Effects of iron overload on estrous cycle and fertility. ((**a**) Microscopic images of vaginal smears from mice at different stages of the estrous cycle. The red scale bar represents 500 μm. (**b**) Statistical graphs of the estrous cycles of three mice in the control group and the iron overload group. The numbers 1, 2, 3, and 4 represent diestrus, proestrus, estrus, and metestrus, respectively. (**c**) Pregnancy rates of the two groups. (**d**) Average litter sizes of the two groups. The experiment was repeated nine times, and data are presented as mean ± standard deviation. One-Way ANOVA was used to compare differences among groups. Significance is indicated as * *p* < 0.05, and ** *p* < 0.01. A difference with *p* < 0.05 was considered statistically significant.).

As shown in Figure 2a, the composition of vaginal secretions varies across different stages of the estrous cycle. During diestrus, the secretions are composed primarily of neutrophils; in proestrus, they mainly consist of epithelial cells; in estrus, they are predominantly cornified epithelial cells; and in metestrus, they feature a mixture of cornified epithelial cells and neutrophils.

From each group, three mice were randomly selected to generate estrous cycle graphs. As shown in Figure 2b, the control group exhibited regular estrous cycle patterns, with each cycle typically lasting 5–6 days. In contrast, the iron-overloaded group showed varying degrees of estrous cycle disruption.

The two groups of mice were then allowed to mate naturally with normal males, and their pregnancy rates and litter sizes were recorded. As shown in Figure 2c,d, both the pregnancy rate (control vs. iron overload: 88.90 ± 7.86% vs. 44.44 ± 19.25%, *p* < 0.05) and the average number of pups born per litter (control vs. iron overload: 9.89 ± 2.22 vs. 3.33 ± 0.33, *p* < 0.01) were significantly lower in the iron overload group than in the control group.

These findings indicate that ovarian iron overload disrupts the estrous cycle in female mice, thereby reducing their reproductive capacity.

### 3.3. Ovarian Iron Overload Reduced the Capacity for Estrogen Synthesis and Follicular Development in Female Mice

**Figure 3:**
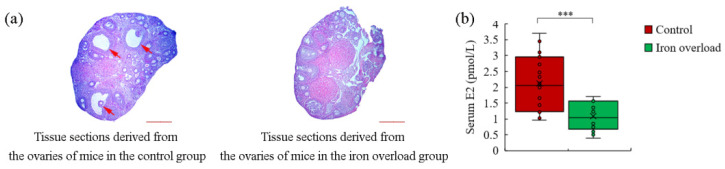
Effects of iron overload on follicle development and serum estradiol levels in female mice. ((**a**) Representative ovarian tissue sections of mice in the iron overload and control groups. Red arrows indicate antral follicles. Scale bar = 500 μm. (**b**) Serum estradiol levels in the two groups. The experiment was repeated ten times, and data are presented as mean ± standard deviation. One-Way ANOVA was used to compare differences among groups. Significance is indicated as *** *p* < 0.001. A difference with *p* < 0.05 was considered statistically significant.).

To further investigate the mechanisms underlying the reduced reproductive capacity caused by ovarian iron overload in female mice, histological sectioning was performed to assess the effects of iron overload on ovarian follicular development. As shown in Figure 3a, compared with the control group, the ovarian tissue in the iron overload group displayed a loose structure with numerous cavity-like formations. More importantly, the ovaries of the control group contained abundant developing antral follicles and even mature follicles (indicated by red arrows in Figure 3a), whereas antral follicles were rarely observed in the iron overload group. These findings suggest that impaired follicular development may represent one of the mechanisms by which iron overload reduces reproductive capacity in female mice.

Granulosa cells within ovarian follicles are the primary site of estrogen synthesis [20]. To investigate whether the arrest of follicular development induced by iron overload affects ovarian estrogen (E2) synthesis in female mice, serum E2 levels were measured in both the control and iron overload groups. As expected, although E2 levels varied considerably among individual mice, the mean serum E2 level in the iron overload group was significantly lower than that in the control group (*p* < 0.001) (Figure 3b).

Together, these findings indicate that iron overload leads to arrested follicular development in the ovaries of female mice, which in turn reduces estrogen synthesis and disrupts the estrous cycle, ultimately diminishing reproductive capacity.

### 3.4. Ovarian Iron Overload Induces Oxidative Stress and Ferroptosis in Granulosa Cells

Granulosa cells are critical for follicular development and female reproductive capacity [20]. To explore the molecular mechanism underlying ovarian iron overload-induced follicular development arrest in mice, granulosa cells were freshly isolated from the control and iron-overloaded groups, and liquid chromatography-mass spectrometry (LC-MS) was used to assess the impact of iron overload on granulosa cell metabolism.

**Figure 4:**
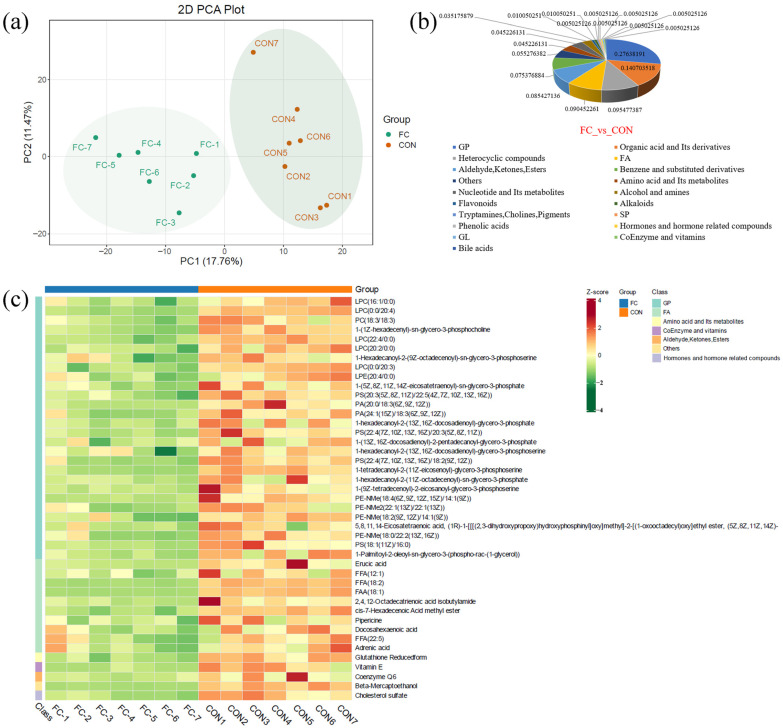
Effects of iron overload on the metabolism of mouse granulosa cells. (Granulosa cells were freshly isolated from the control and iron overload groups. Liquid chromatography-mass spectrometry (LC-MS) was used to assess the impact of iron overload on granulosa cell metabolism. (**a**) Principal component analysis (PCA) of differential metabolites. (**b**) Categories and proportions of differential metabolites. (**c**) Heatmap of selected differential metabolites.).

A total of 1264 metabolites were detected across the two groups, of which 1048 were identified at level 2 (639 T3-positive and 409 T3-negative metabolites) (Table 3). PCA was performed to visualize the overall distribution of metabolic profiles among the samples. The PCA score plot (Figure 4a) showed a clear separation between the iron overload and control groups along the first principal component (PC1), which accounted for 17.76% of the total variance; the second principal component (PC2) explained 11.47% of the variance. The two groups formed distinct clusters without overlap, indicating that iron overload induced a systematic alteration in the metabolic landscape.

A total of 199 significantly differential metabolites were identified between the two groups (VIP > 1 and *p* < 0.05, Student’s *t*-test). Among these, 107 metabolites were significantly downregulated and 92 significantly upregulated in the iron overload group (Table 3). GPs accounted for the largest proportion of these differential metabolites (27.64%), followed by organic acids and their derivatives (14.07%) (Figure 4b).

Further analysis of the significantly differential metabolites revealed that 74.55% (41/55) of GPs and 77.78% (14/18) of fatty acids (FAs) contained unsaturated bonds. Among these, 68.29% (28/41) of unsaturated bond-containing GPs and 71.43% (10/14) of unsaturated bond-containing FAs were significantly downregulated in the iron overload group, including phosphatidylcholine (PC) (18:3/18:3), lysophosphatidylcholine (LPC) (20:4/0:0), and free fatty acid (FFA) (18:2) (Figure 4c). In addition, the levels of several antioxidants, including GSH, VE, and CoQ6, were significantly decreased in the iron overload group (Figure 4c). These results indicate that iron overload induces oxidative stress in granulosa cells, leading to depletion of intracellular antioxidants and disruption of unsaturated bond-containing GPs and FAs, thereby resulting in a significant reduction in these substances.

**Figure 5:**
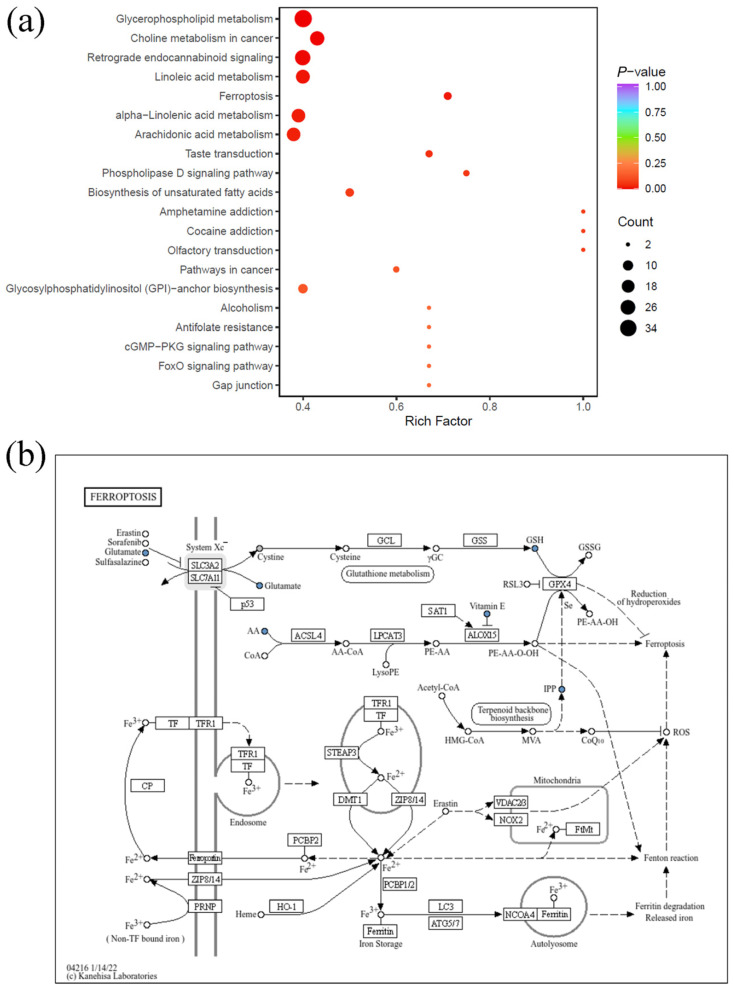
KEGG pathway enrichment analysis of differential metabolites between two groups of cells. ((**a**) KEGG pathway enrichment analysis of differential metabolites. (**b**) Differential metabolites enriched in the ferroptosis signaling pathway. Blue dots indicate metabolites significantly downregulated in the iron overload group.).

KEGG pathway enrichment analysis showed that the differential metabolites between the two groups were mainly enriched in pathways such as glycerophospholipid metabolism and ferroptosis (Figure 5a). In the ferroptosis signaling pathway, the significantly decreased levels of GSH, VE, isopentenyl diphosphate (IPP), and glutamate indicated that ovarian iron overload induced ferroptosis in granulosa cells (Figure 5b).

In summary, ovarian iron overload induces oxidative stress in granulosa cells, leading to antioxidant depletion, disruption of unsaturated GPs and FAs, and ultimately ferroptosis, which may underlie the reduced estrogen synthesis and diminished reproductive capacity in mice.

### 3.5. Activation of the Steroid Synthesis Pathway in Iron-Overloaded Granulosa Cells

**Figure 6:**
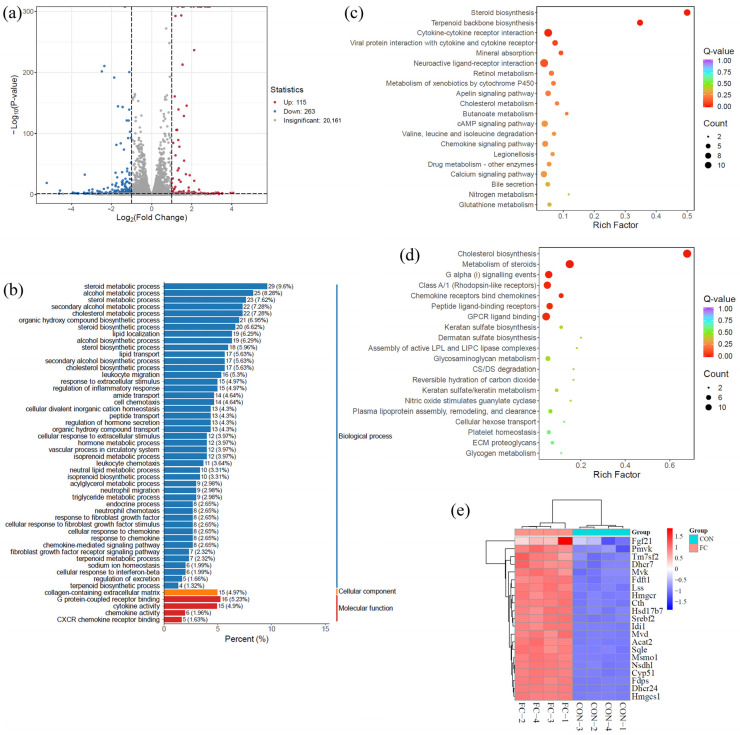
Effects of iron overload on gene expression in mouse granulosa cells. (Granulosa cells were freshly isolated from the control and iron overload groups. RNA sequencing was used to examine the impact of iron overload on gene expression in granulosa cells. (**a**) Volcano plot of differentially expressed genes between the control and iron overload groups. (**b**) GO enrichment analysis of differentially expressed genes. (**c**) KEGG pathway enrichment analysis of differentially expressed genes. (**d**) Reactome enrichment analysis of differentially expressed genes. (**e**) Heatmap of selected differentially expressed genes.).

RNA sequencing was employed to investigate the impact of iron overload on gene expression in granulosa cells. As shown in Figure 6a, a total of 20,539 genes were detected across the two groups. Among these, 115 genes were significantly upregulated and 263 genes were significantly downregulated in the iron overload group (|log_2_(fold change)| > 1 and *p* < 0.05).

**Figure 7:**
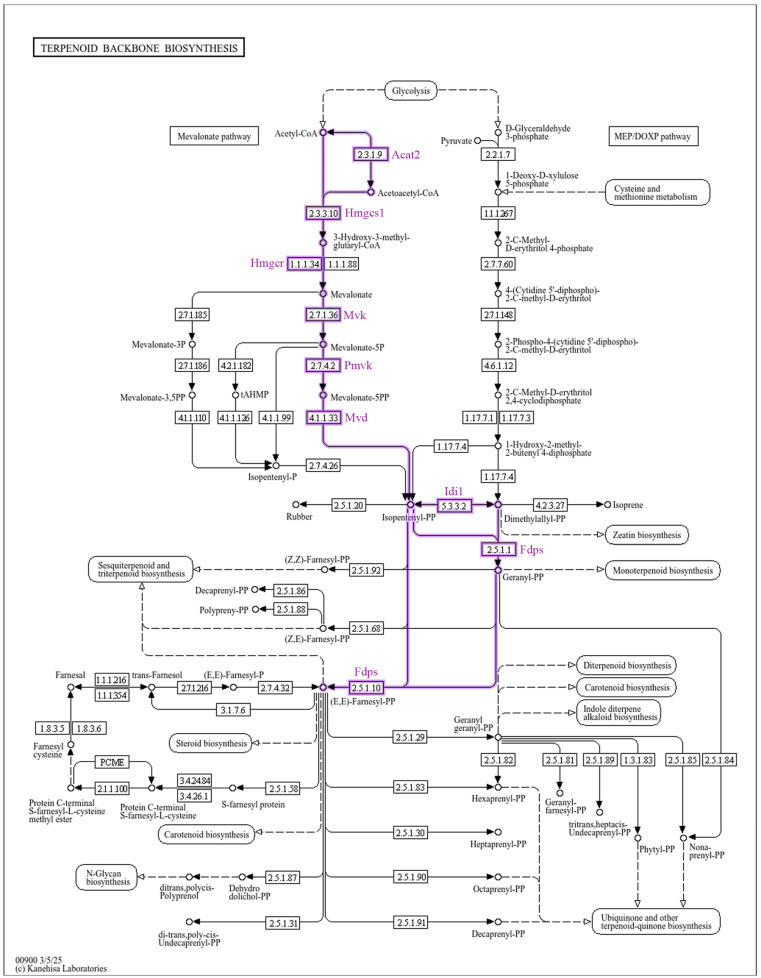
Differentially expressed genes enriched in the terpenoid backbone biosynthesis pathways. (Cyan boxes indicate genes significantly upregulated in the iron overload group.).

**Figure 8:**
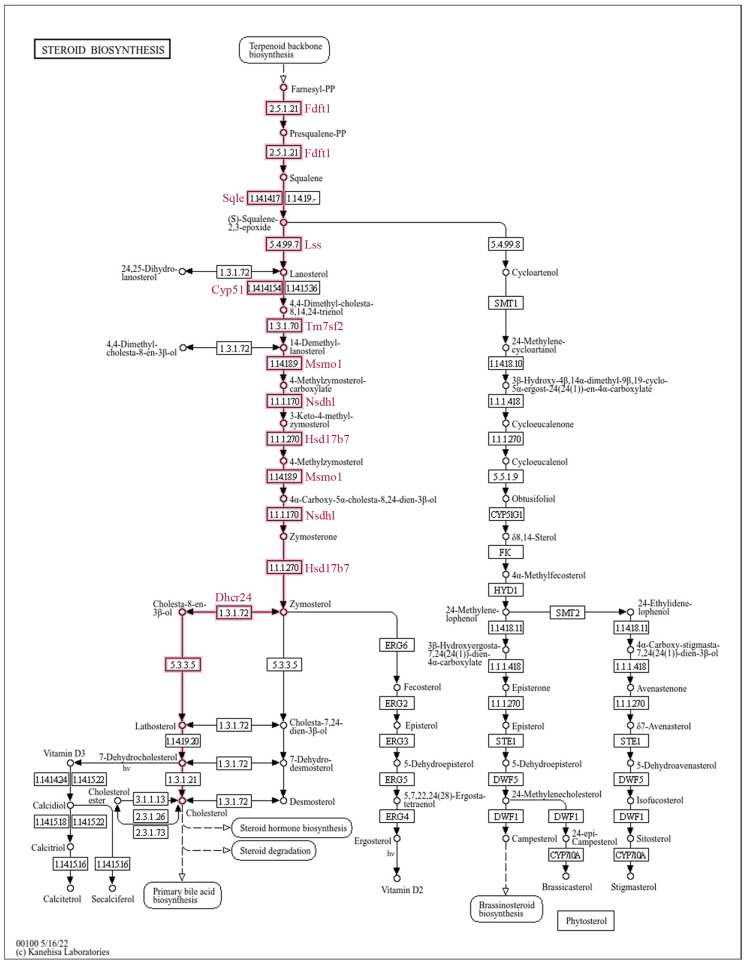
Differentially expressed genes enriched in the steroid biosynthesis pathways. (Red boxes indicate genes significantly upregulated in the iron overload group.).

GO, KEGG, and Reactome enrichment analyses of differentially expressed genes all converged on steroid synthesis-related signaling pathways, including steroid metabolic process, steroid biosynthesis, terpenoid backbone biosynthesis, cholesterol biosynthesis, and metabolism of steroids (Figure 6b–d). Notably, terpenoid backbone biosynthesis is an upstream step of steroid biosynthesis. Further analysis revealed that, in the iron overload group, the expression levels of multiple genes encoding enzymes involved in the pathway from acetyl-CoA to cholesterol were significantly upregulated, including *Hmgcr*, which encodes the rate-limiting enzyme of the cholesterol synthesis pathway (Figure 6e, Figure 7 and Figure 8). SREBF2 is a master transcription factor that regulates the expression of most key enzyme-encoding genes in the steroid synthesis pathway [25,26,27]. The expression level of *Srebf2* was significantly upregulated in the iron overload group (Figure 6e), which may explain the marked upregulation of multiple enzyme genes in the steroid synthesis pathway observed in iron-overloaded granulosa cells.

Several intermediates in the steroid synthesis pathway, including IPP, squalene, coenzyme Q10 (CoQ10), and 7-dehydrocholesterol (7-DHC), possess direct or indirect antioxidant or anti-ferroptotic effects [28,29,30,31,32]. Therefore, activation of the steroid synthesis pathway by SREBF2 in iron-overloaded granulosa cells may promote the synthesis of these antioxidants or anti-ferroptotic intermediates, thereby inhibiting ferroptosis. This hypothesis is further supported by the metabolomic findings of decreased levels of the steroid synthesis intermediates IPP and CoQ6, as well as the end product cholesterol (Figure 4c). However, the mechanism underlying the upregulation of *Srebf2* expression in iron-overloaded granulosa cells requires further investigation.

### 3.6. Iron Overload Induces Ferroptosis and Activates the Steroid Synthesis Pathway in Cultured Ovarian Granulosa Cells In Vitro

**Figure 9:**
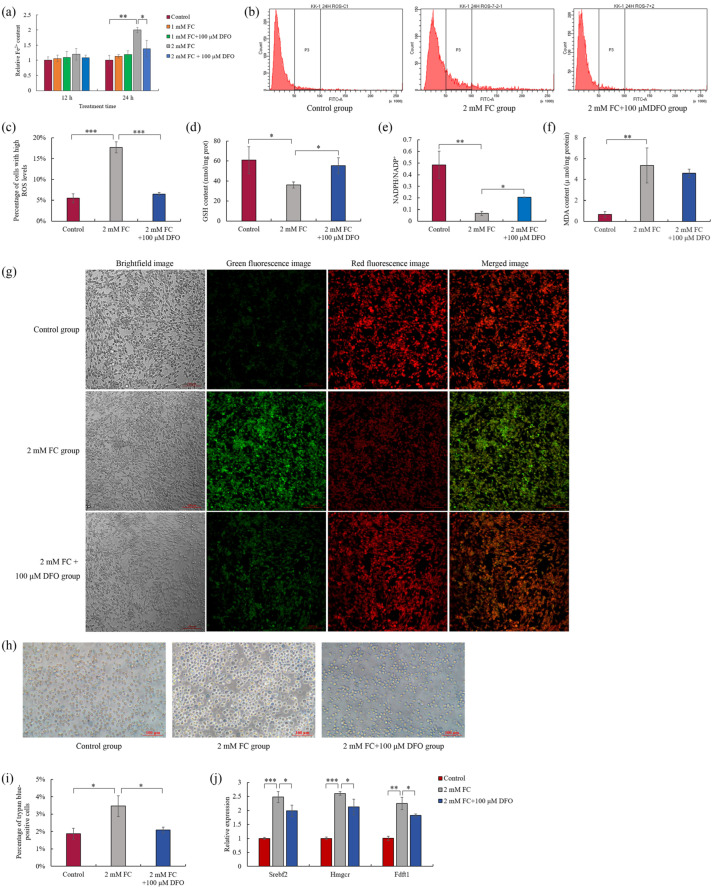
Iron overload induces ferroptosis and activates steroidogenesis in KK1 cells. ((**a**) Fe^2+^ content in cultured mouse granulosa cells (KK1) after treatment with 1 mM or 2 mM ferric citrate (FC) alone or in combination with 100 μM deferoxamine (DFO) for 12 h and 24 h. (**b**) Cells from the three groups (control, 2 mM FC alone for 24 h, or 2 mM FC combined with 100 μM DFO treatment for 24 h) were stained with the reactive oxygen species (ROS) fluorescent probe DCFH-DA, and their fluorescence intensity was measured by flow cytometry. (**c**) Percentage of cells with high ROS levels (cells in P3 gate in (**b**)) in the above three groups. (**d**) Glutathione (GSH) content in the three groups. (**e**) NADPH/NADP^+^ ratio in the three groups. (**f**) Malondialdehyde (MDA) content in the three groups. (**g**) Representative images of lipid peroxidation detected by the BDPY 581/591 C11 probe in the three groups. Brightfield images, green fluorescence (oxidized state, excitation 488 nm/emission 510 nm), red fluorescence (reduced state, excitation 581 nm/emission 591 nm), and merged images are shown. Red scale bar = 100 μm. (**h**) Representative microscopic images of cells from the three groups after Trypan blue staining. Scale bar = 100 μm. (**i**) Percentage of Trypan blue-positive cells in the three groups. (**j**) Relative mRNA levels of *Hmgcr*, *Fdft1*, and *Srebf2* in the three groups were determined by qRT-PCR. The experiment was repeated three times, and data are presented as mean ± standard deviation. One-Way ANOVA was used to compare differences among groups. Significance is indicated as * *p* < 0.05, ** *p* < 0.01, and *** *p* < 0.001. A difference with *p* < 0.05 was considered statistically significant.).

To further verify that iron overload induces ferroptosis in granulosa cells, the mouse granulosa cell line KK1 was cultured in vitro and treated with FC, either alone or in combination with the iron chelator DFO. At different time points post-treatment, cellular Fe^2+^ levels were measured using a commercial iron assay kit. As shown in Figure 9a, treatment with 1 mM or 2 mM FC for 12 h did not lead to a significant increase in intracellular Fe^2+^ levels in KK1 cells. However, treatment with 2 mM FC for 24 h significantly elevated intracellular Fe^2+^ levels (*p* < 0.01). Thus, KK1 cells treated with 2 mM FC for 24 h met the predefined criterion for the iron-overloaded cellular model and were used for subsequent experiments. Furthermore, 100 μM DFO markedly attenuated the FC-induced elevation of intracellular Fe^2+^ levels.

Three groups of cells (control, 2 mM FC, and 2 mM FC combined with 100 μM DFO treatment for 24 h) were stained with the ROS fluorescent probe DCFH-DA, and the fluorescence intensity was measured by flow cytometry. As shown in Figure 9b,c, the proportion of cells with elevated ROS levels (cells in the P3 gate) in the 2 mM FC treatment group was significantly higher than that in the control group (*p* < 0.001). Moreover, DFO significantly reduced the FC-induced increase in ROS levels in granulosa cells.

Subsequent measurements showed that oxidative stress induced by iron overload significantly decreased intracellular GSH content and the NADPH/NADP^+^ ratio in granulosa cells (Figure 9d,e). These findings were consistent with the metabolomics results, demonstrating that the oxidative stress caused by iron overload substantially consumed intracellular antioxidants, leading to their reduced levels.

MDA is an oxidation product of polyunsaturated fatty acids (PUFAs) [33,34]. As shown in Figure 9f, iron overload significantly increased the MDA content in granulosa cells (*p* < 0.05). This finding indicates that the reduced levels of GPs and FAs containing polyunsaturated bonds observed in the metabolomics analysis are attributable to their oxidation.

Lipid peroxidation is one of the cornerstones in the homeostasis control of ferroptosis [35]. A lipid peroxidation probe, BDPY 581/591 C11, was employed to assess the degree of lipid peroxidation in the three groups of cells. Under the oxidized state, this probe emitted green fluorescence at 510 nm upon excitation at 488 nm, whereas in the reduced state, it emitted red fluorescence at 591 nm upon excitation at 581 nm. As shown in Figure 9g, the green fluorescence intensity in the 2 mM FC-treated group was significantly higher than that in the control group, while the red fluorescence intensity was markedly lower. Moreover, in the group treated with 2 mM FC combined with 100 μM DFO, both green and red fluorescence intensities were intermediate between those of the control group and the FC treatment group. These results indicate that iron overload can induce lipid peroxidation in granulosa cells.

Lipid peroxidation-induced plasma membrane rupture is a major cause of ferroptosis [36]. Trypan blue staining was used to assess cell membrane integrity. As shown in Figure 9h,i, the percentage of trypan blue-positive cells in iron-overloaded granulosa cells was significantly higher than that in the control group (*p* < 0.05), indicating that iron overload induces ferroptosis in granulosa cells. This finding is consistent with the significant enrichment of the ferroptosis signaling pathway observed in the metabolomics analysis.

qRT-PCR was performed to assess the effects of iron overload on the expression levels of the enzyme genes in the steroid synthesis pathway in mouse granulosa cells. As shown in Figure 9j, the expression levels of *Hmgcr* and *Fdft1* were significantly upregulated in the 2 mM FC-treated group, whereas 100 μM DFO markedly alleviated this upregulation. Furthermore, iron overload led to a significant upregulation of *Srebf2*, the major transcription factor for enzyme genes in the steroid synthesis pathway. These results were consistent with the RNA-sequencing data, further indicating that iron overload causes a marked upregulation of *Srebf2* expression in granulosa cells, which in turn upregulates the expression of multiple enzyme genes in the steroid synthesis pathway and activates this signaling pathway.

### 3.7. Knockdown of Srebf2 Exacerbates Ferroptosis in Iron-Overloaded Granulosa Cells

**Figure 10:**
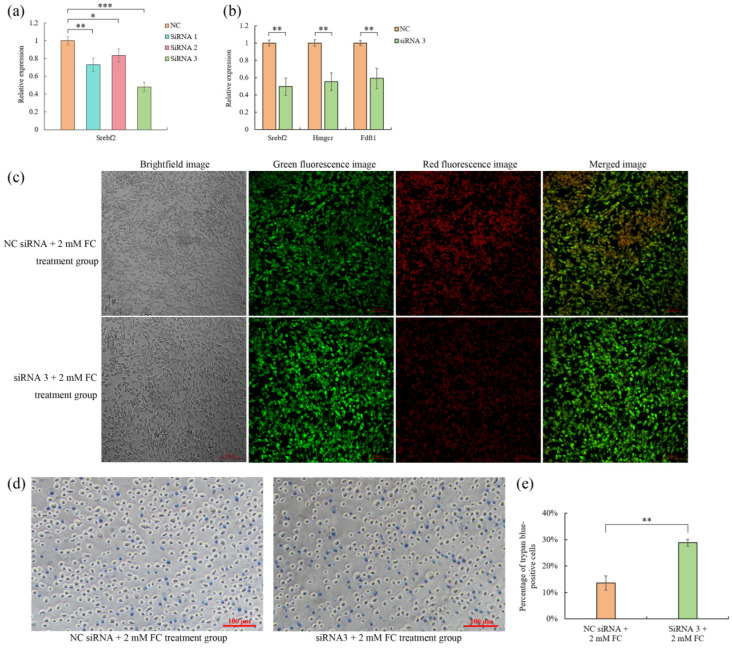
*Srebf2* knockdown exacerbates ferroptosis in iron-overloaded KK1 cells. ((**a**) KK1 cells were transfected with negative control (NC) siRNA or three pairs of *Srebf2*-specific siRNAs for 36 h, after which the knockdown efficiency of the three siRNAs was assessed by qRT-PCR. (**b**) Following *Srebf2* knockdown using siRNA 3, the expression levels of *Hmgcr* and *Fdft1* were examined by qRT-PCR. (**c**) At 12 h post-transfection, cells were treated with 2 mM ferric citrate (FC) for 24 h. A BDPY 581/591 C11 lipid peroxidation detection kit was used to measure the degree of lipid peroxidation. (**d**) Trypan blue staining was employed to evaluate the effect of *Srebf2* knockdown on cell membrane integrity. (**e**) Percentage of Trypan blue-positive cells in the two groups. The experiment was repeated three times, and data are presented as mean ± standard deviation. One-Way ANOVA was used to compare differences among groups. Significance is indicated as * *p* < 0.05, ** *p* < 0.01, and *** *p* < 0.001. A difference with * *p* < 0.05 was considered statistically significant.).

To further confirm the activation role of *Srebf2* in the steroid synthesis pathway, three pairs of synthetic *Srebf2*-specific siRNAs (see Table 2) and a pair of NC siRNA were transfected into cultured KK1 cells using Lipo8000. At 36 h post-transfection, qRT-PCR was performed to evaluate the silencing specificity of the three siRNAs. As shown in Figure 10a, although all three siRNAs significantly knocked down *Srebf2* mRNA levels, siRNA 3 exhibited the strongest knockdown efficiency, reducing *Srebf2* expression to 48.0 ± 5.3% of that in the NC siRNA-transfected group (*p* < 0.001). Subsequently, qRT-PCR was used to examine the effects of *Srebf2* knockdown on the expression of *Hmgcr* and *Fdft1*, two rate-limiting enzymes in the steroid synthesis pathway. The results showed that knockdown of *Srebf2* using siRNA 3 also significantly decreased the expression levels of *Hmgcr* and *Fdft1* (Figure 10b), confirming the activating role of *Srebf2* in the steroid synthesis pathway.

To further investigate the effect of SREBF2-mediated activation of the steroid synthesis pathway on lipid peroxidation and ferroptosis in iron-overloaded granulosa cells, NC siRNA and siRNA 3 were transfected into cells for 12 h, followed by treatment with 2 mM FC for 24 h. Lipid peroxidation levels and cell membrane integrity were assessed using a lipid peroxidation assay kit and Trypan blue staining, respectively. As shown in Figure 10c, the green fluorescence intensity was markedly higher in the siRNA 3-transfected group than that in the NC siRNA-transfected group, indicating that following *Srebf2* knockdown and the consequent suppression of the steroid synthesis pathway, iron overload-induced lipid peroxidation was significantly exacerbated. Moreover, the proportion of Trypan blue-positive cells was also significantly (*p* < 0.01) increased after *Srebf2* knockdown (Figure 10d,e), further demonstrating that interference with SREBF2-mediated activation of the steroid synthesis pathway leads to increased intracellular lipid peroxidation and exacerbates membrane rupture and ferroptosis.

The above results indicate that interference with SREBF2-mediated activation of the steroid synthesis pathway exacerbates ferroptosis in iron-overloaded granulosa cells, inversely validating that the steroid synthesis pathway serves as an anti-ferroptosis pathway. However, the mechanism underlying the upregulation of *Srebf2* expression in iron-overloaded granulosa cells requires further investigation.

## 4. Discussion

Iron is an essential trace element, but its excess promotes the generation of ROS via the Fenton reaction, overwhelming the cellular antioxidant capacity and triggering oxidative stress [9,10,11,12,13]. In the ovary, iron overload has been shown to compromise folliculogenesis, oocyte quality, and steroidogenesis [5,6,14,15,16,17,18,19]. However, the precise mode of cell death responsible for these deleterious effects remains incompletely understood. This study provides multiple lines of evidence that ferroptosis—an iron-dependent, lipid peroxidation-driven form of regulated cell death—is a key mechanism in iron-overloaded granulosa cells.

Metabolomic analysis revealed a significant depletion of unsaturated GPs and FAs, along with reduced levels of antioxidants such as VE, and CoQ6. These changes are hallmarks of ferroptosis, as the peroxidation of PUFAs consumes GSH and other radical-scavenging molecules [28,33,34]. In addition, increased MDA content, a stable end product of PUFAs peroxidation, was observed. Elevated trypan blue staining, indicative of plasma membrane rupture—the final execution step of ferroptosis [36]—was also detected. Consistently, the ferroptosis signaling pathway was significantly enriched in KEGG analysis of the differential metabolites. The in vitro experiments further confirmed that FC treatment elevated intracellular Fe^2+^ and ROS levels, decreased GSH and the NADPH/NADP^+^ ratio, and increased lipid peroxidation and membrane damage. Collectively, these results establish that iron overload directly triggers ferroptosis in granulosa cells.

Ferroptosis of granulosa cells inevitably leads to follicular arrest. Granulosa cells are essential for follicle survival and growth, as they provide nutrients, growth factors, and steroid hormones [20]. Their ferroptotic death results in a reduced number of antral follicles, diminished estrogen synthesis, disruption of the estrous cycle, and ultimately lower pregnancy rates and litter size. In this study, histological data showing scarce antral follicles in iron-overloaded ovaries, together with the marked decrease in serum E2, support this causal chain. These findings align with clinical observations that iron-overloaded women with transfusion-dependent β-thalassemia major exhibit reduced antral follicle counts, diminished ovarian reserve, and subfertility [5,6,17,18]. Thus, therapeutic targeting ferroptosis may represent a promising strategy to preserve fertility in conditions of iron overload.

In the present study, a surprising finding from transcriptomic analysis was the significant upregulation of multiple genes encoding enzymes in the steroid biosynthesis pathway, including *Hmgcr* (the rate-limiting enzyme of cholesterol synthesis) and *Fdft1*, in iron-overloaded granulosa cells. This upregulation was accompanied by increased expression of *Srebf2*, the master transcription factor that controls the expression of most steroidogenic enzyme genes [25,26,27]. Given that the final product of this pathway, estrogen, was reduced in the serum, it seemed paradoxical that the pathway was transcriptionally activated. However, several intermediates of the cholesterol biosynthetic pathway–such as IPP, squalene, CoQ10 (ubiquinone), and 7-DHC—possess documented antioxidant or direct anti-ferroptotic activities [28,29,30,31,32]. For instance, squalene accumulation in cholesterol-auxotrophic lymphomas prevents oxidative cell death [30]; 7-DHC has recently been identified as an endogenous suppressor of ferroptosis that operates by trapping lipid peroxyl radicals [31,32]; and CoQ10 acts as a lipophilic radical scavenger and also recycles other antioxidants [29]. IPP, as a building block for the synthesis of coenzyme Q and other isoprenoids, indirectly contributes to the cellular defense against lipid peroxidation.

In the metabolomic data from the present study, despite the increased expression of synthesis genes, the levels of IPP, CoQ6, and cholesterol were significantly lower in iron-overloaded granulosa cells compared to controls. It is proposed that two converging mechanisms account for this apparent paradox. First, the protective intermediates of the steroid biosynthesis pathway (IPP, coenzyme Q, and 7-DHC) are continuously consumed to counteract lipid peroxidation, with their consumption rate outstripping the upregulated synthetic capacity. Second, the synthesis of cholesterol and steroid hormones is dependent on adequate NADPH, cytosolic acetyl-CoA, and ATP. In iron-overloaded cells, a substantial portion of NADPH is diverted to regenerate GSH and other antioxidant systems, leaving insufficient reducing equivalents to sustain the reductive steps of steroidogenesis. This NADPH competition, combined with the accelerated consumption of protective intermediates, explains why transcriptional activation of the upstream cholesterol synthesis pathway fails to restore estrogen levels and ultimately results in ferroptosis. When *Srebf2* was knocked down with siRNA, the expression of *Hmgcr* and *Fdft1* was reduced, and more importantly, lipid peroxidation and ferroptosis were exacerbated, as evidenced by increased green fluorescence of the BODIPY 581/591 C11 probe and a higher percentage of trypan blue-positive cells. These results provide direct causal evidence that the SREBF2-driven steroid synthesis pathway acts as an endogenous anti-ferroptotic mechanism. To our knowledge, this is the first demonstration that the canonical steroid biosynthesis pathway can be activated to counteract ferroptosis, shifting the traditional view of this pathway from merely producing steroid hormones to also serving as a metabolic shield against oxidative lipid damage. It is therefore proposed that the steroid biosynthesis pathway is a bona fide anti-ferroptosis pathway, at least in granulosa cells.

The recognition that the steroid biosynthesis pathway can suppress ferroptosis has broad implications. In the context of female reproduction, activation of this pathway represents an endogenous attempt to maintain granulosa cell survival under iron overload stress. Enhancing the production of anti-ferroptotic intermediates (e.g., by supplying squalene, CoQ10, or 7-DHC precursors) could be a novel therapeutic strategy to preserve ovarian function in iron overload-related infertility, such as in thalassemia major, hereditary hemochromatosis, or occupational iron exposure [2,3,4,5,19]. Conversely, in oncology, many cancer cells exhibit elevated cholesterol synthesis to support membrane biogenesis and proliferation [27]. The present findings raise the possibility that the steroid biosynthesis pathway may also protect cancer cells from ferroptosis, which is increasingly recognized as a mechanism of action for certain chemotherapeutic agents and for immunotherapy [28,35]. Therefore, pharmacological inhibition of SREBF2 or downstream enzymes (e.g., HMGCR by statins) could sensitize cancer cells to ferroptosis-inducing therapies. Indeed, statins have been reported to enhance ferroptosis in some tumor models [28,35]. The dual role of this pathway—beneficial in normal cells but potentially deleterious in cancer—underscores its potential as a therapeutic target that requires context-specific modulation.

The mechanism by which iron overload upregulates *Srebf2* expression in granulosa cells remains incompletely defined. It is hypothesized that iron-induced oxidative stress and lipid peroxidation may trigger a feedback loop that activates SREBF2. One plausible route is the unfolded protein response (UPR) of the endoplasmic reticulum (ER). Iron overload promotes ROS generation, which can disturb ER homeostasis and induce ER stress [9,14]. ER stress is known to activate the sterol regulatory element-binding protein (SREBP) pathway through the ER-resident protein Insig and Scap [27]. Alternatively, the depletion of cholesterol and certain oxysterols in the ER membrane, which could result from increased consumption or oxidation of cholesterol intermediates, may directly relieve the inhibition of Scap, allowing its escort of SREBP to the Golgi for proteolytic activation [26,27]. Moreover, lipid peroxidation products such as 4-hydroxynonenal (4-HNE) have been shown to modulate transcription factor activity [34]. Whether these products directly affect *Srebf2* transcription or the post-translational processing of SREBF2 requires further investigation. In addition, other transcription factors such as NF-κB or Nrf2, which are redox-sensitive, could potentially cross-talk with the SREBP pathway. Future studies employing ROS scavengers (e.g., N-acetylcysteine), ER stress inhibitors (e.g., 4-phenylbutyrate), or specific knockdown of upstream regulators are needed to dissect the precise signaling cascade linking iron overload to SREBF2 activation.

A limitation of this study is that E2 secretion was not measured in the KK1 cell culture medium. While in vivo data support the link between iron overload and reduced estrogen synthesis, direct measurement of E2 in the in vitro system would further establish the functional consequence of ferroptosis on steroidogenesis. This represents a limitation of the present study and warrants future investigation.

## 5. Conclusions

**Figure 11:**
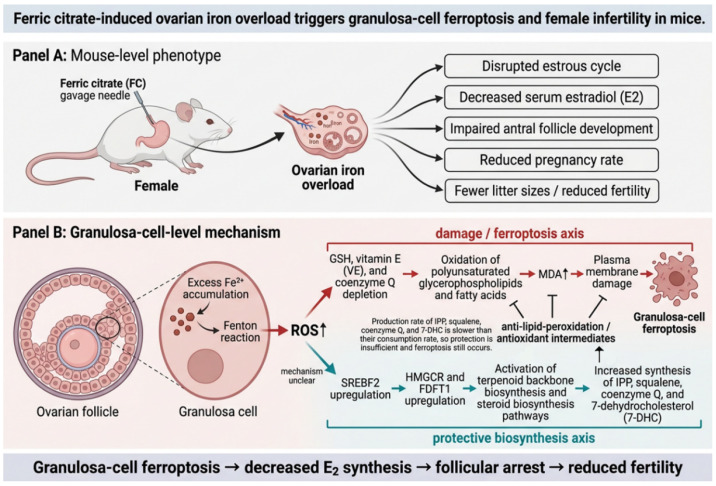
The proposed mechanism by which iron overload impairs female reproductive capacity in mice.

In conclusion, this study demonstrates that excessive iron intake induces ovarian iron overload in mice, leading to disrupted estrous cycles, a marked reduction in serum E2 levels, impaired follicular development, and ultimately decreased pregnancy rates and litter sizes. Mechanistically, iron overload in mouse granulosa cells triggers the Fenton reaction, resulting in elevated intracellular ROS levels. On one hand, increased ROS promotes ferroptosis through the following cascade: depletion of GSH, VE, and coenzyme Q, which leads to oxidation of GPs and FAs containing polyunsaturated bonds. This oxidation subsequently elevates MDA levels and ultimately causes plasma membrane damage and the occurrence of ferroptosis. On the other hand, ROS upregulates *Srebf2* expression via mechanisms yet to be elucidated, which in turn enhances the expression of multiple enzyme-encoding genes in the terpenoid backbone biosynthesis and steroid biosynthesis pathways, including *Hmgcr* and *Fdft1*. Activation of these two pathways increases the synthesis of protective intermediates such as IPP, squalene, coenzyme Q, and 7-DHC, which counteract lipid peroxidation and ferroptosis. However, because the generation rate of these protective intermediates is slower than their consumption rate, ferroptosis in granulosa cells eventually occurs, manifesting as reduced estrogen levels, arrested follicular development, and diminished reproductive capacity in mice (Figure 11). These findings provide a comprehensive mechanistic understanding of iron overload-induced female infertility and highlight the steroid biosynthesis pathway as a potential therapeutic target.

## Figures and Tables

**Table 1:** The primers for qRT-PCR.

Primer	Sequence	Tm (°C)	Product Length (bp)
Srebf2 forward primer	5′-ACCCCTTGACTTCCTTGCTG-3′	59.89	282
Srebf2 reverse primer	5′-CACCTTTGGCGAGGTCTAGG-3′	60.11	
Hmgcr forward primer	5′-CACGCTCATAGTCGCTGGAT-3′	59.97	276
Hmgcr reverse primer	5′-CACGACGGGAGACGTGATAG-3′	59.97	
Fdft1 forward primer	5′-AGAAGGACCGACAAGTGCTG-3′	59.97	159
Fdft1 reverse primer	5′-CCCAGTCCTGTTTGGAGGTC-3′	59.96	
Tubulin forward primer	5′-TTCTGGTGGACTTGGAACCTG-3′	59.86	176
Tubulin reverse primer	5′-TTTCCGCACGACATCTAGGA-3′	59.82	

**Table 2:** Sequences of siRNA against *Srebf2* and NC siRNA.

siRNA	Strand	Sequence
siRNA1 against Srebf2	sense strand	5′-CAUUGAUUACAUCAAAUUAUCU-3′
	antisense strand	5′-AUAUUUGAUGUAAUCAAUGGC-3′
siRNA2 against Srebf2	sense strand	5′-GAAAUUACUUCAUUCUUUUGU-3′
	antisense strand	5′-AAAAGAAUGAAGUAAUUUCAA-3′
siRNA3 against Srebf2	sense strand	5′-GUUUCAUUCCAGUUUUUGUUU-3′
	antisense strand	5′-ACAAAAACUGGAAUGAAACGU-3′
NC siRNA	sense strand	5′-UUCCUCCAACGUGUCACGUTT-3′
	antisense strand	5′-ACGUGACACGUUCGGAGAATT-3′

**Table 3:** Numbers of total and differential metabolites detected in the iron overload and control groups.

Group Name	All Compounds	All Insignificance	All Significant Difference	Down-Regulated	Up-Regulated
FC_vs_CON	1048	849	199	107	92

Note: The FC and CON groups refer to the iron overload group treated with FC and the control group treated with saline, respectively.

## Data Availability

The raw data used to support the findings of this study are deposited in the Zenodo database and are publicly available at: https://doi.org/10.5281/zenodo.21258264.

## Supplementary Materials

The following supporting information can be downloaded at: https://www.mdpi.com/article/10.3390/biology15141182/s1, Figure S1: Schematic overview of the in vivo experimental design.

## Abbreviations

The following abbreviations are used in this manuscript:

ANOVA	Analysis of variance
CoQ6	Coenzyme Q6
CoQ10	Coenzyme Q10
CV	Coefficient of Variation
DCFH-DA	2′,7′-Dichlorofluorescein diacetate
DFO	Deferoxamine
DMEM	Dulbecco’s modified Eagle’s medium
E2	Estradiol
ELISA	Enzyme-linked immunosorbent assay
ER	Endoplasmic reticulum
FAs	Fatty acids
FBS	Fetal bovine serum
FFA	Free fatty acid
FC	Ferric citrate
GO	Gene Ontology
GPs	Glycerophospholipids
GSH	Glutathione
HE	Hematoxylin and eosin
IPP	Isopentenyl diphosphate
KEGG	Kyoto Encyclopedia of Genes and Genomes
LC-MS	Liquid chromatography-mass spectrometry
LPC	Lysophosphatidylcholine
MDA	Malondialdehyde
NADP^+^	Nicotinamide adenine dinucleotide phosphate
NADPH	Reduced nicotinamide adenine dinucleotide phosphate
NC	Negative control
PCA	Principal component analysis
PC	Phosphatidylcholine
PMSG	Pregnant mare serum gonadotropin
PUFAs	Polyunsaturated fatty acids
QC	Quality Control
qRT-PCR	Quantitative real-time polymerase chain reaction
Reactome	Reactome Pathway Database
RNAi	RNA interference
ROS	Reactive oxygen species
SD	Standard deviation
siRNA	Small-interfering RNA
SREBP	Sterol regulatory element-binding protein
StAR	Steroidogenic Acute Regulatory Protein
UPR	Unfolded protein response
VE	Vitamin E
VIP	Variable Importance in Projection
4-HNE	4-hydroxynonenal
7-DHC	7-dehydrocholesterol
