# Supplementary material for: Steroid Biosynthesis Pathway Counteracts Iron Overload-Induced Ferroptosis in Mouse Granulosa Cells
Source: Biology (Basel). 2026 Jul 17;15(14):1182. doi: 10.3390/biology15141182 (PMC13405594; doi:10.3390/biology15141182)
Supplement: Supplementary file 1 [file biology-15-01182-s001.zip › biology-4415711-supplementary.pdf]

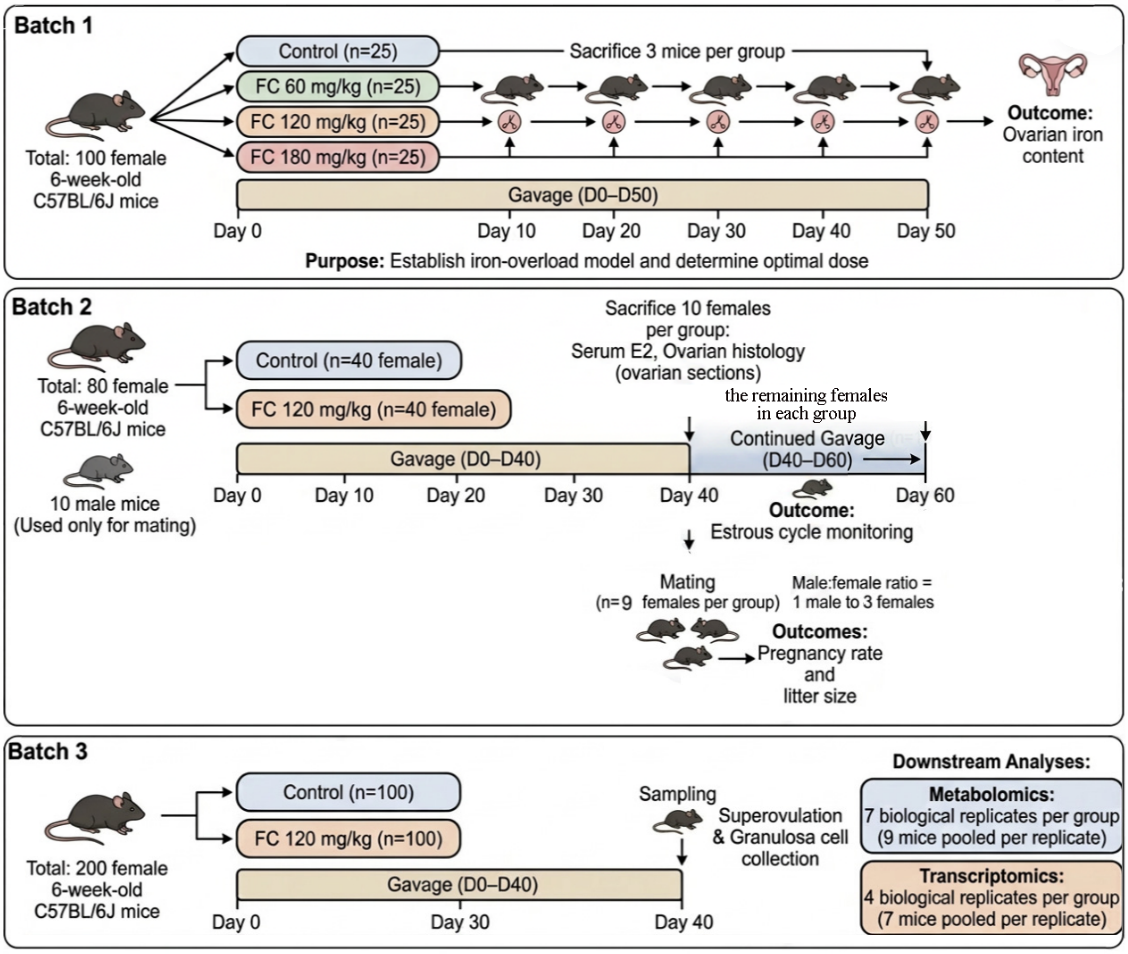

Figure S1. Schematic overview of the in vivo experimental design.

(Female C57BL/6J mice (6 weeks old) were purchased in three separate batches for different experimental purposes. (a) Batch 1 (n = 100): Mice were randomly divided into four groups (n = 25 per group): control (saline) and ferric citrate (FC) treatment groups at doses of 60, 120, or 180 mg/kg/day via oral gavage for up to 50 consecutive days. Every 10 days, three mice from each group were euthanized, and ovarian tissues were collected to measure Fe^{2+} content. This batch was used to establish the iron overload model and determine the optimal FC dose. (b) Batch 2 (n = 80 females + 10 males): Mice were divided into control and FC 120 mg/kg groups (n = 40 per group). After 40 days of gavage, 10 mice per group were euthanized for serum estradiol (E2) measurement and ovarian histology. Another 9 mice per group were co-housed with fertile males (female:male ratio = 3:1) to assess pregnancy rates and litter sizes. The remaining mice continued gavage until day 60 for daily estrous cycle monitoring (from day 41 to day 60). (c) Batch 3 (n = 200): Mice were divided into control and FC 120 mg/kg groups (n = 100 per group). After 40 days of gavage, all mice were superovulated with PMSG, and granulosa cells were isolated 36 h later. For metabolomic analysis, 7 biological replicates were used per group (each replicate pooled from 9 mice). For transcriptomic analysis, 4 biological replicates were used per group (each replicate pooled from 7 mice). To account for potential animal losses during the long-term gavage procedure, all batches were set up with additional mice beyond the numbers strictly required for the downstream assays. Mice that were not used in the specified analyses served as backups and were excluded from the final results.)
